# Individual and structural benefits, challenges, and strategies for integrating medication assistance treatment and antiretroviral services for persons living with HIV who use drugs in Dar es Salaam, Tanzania

**DOI:** 10.21203/rs.3.rs-8346864/v1

**Published:** 2026-02-18

**Authors:** Dorothy Mushi, Peter Sakejo, Magreat Somba, David Huh, Deepa Rao, Sylvia Kaaya

**Affiliations:** Muhimbili University of Health and Allied Sciences; Muhimbili University of Health and Allied Sciences; Ifakara Health Institute; University of Washington; University of Washington; Muhimbili University of Health and Allied Sciences

**Keywords:** medication assistance treatment, persons living with HIV who use drugs, individual and structural challenges and benefits

## Abstract

**Background:**

People who use drugs (PWUDs) have an increased risk of acquiring and transmitting bloodborne diseases, including HIV, hepatitis B and C virus. To manage HIV among people who use drugs and live with HIV (PUD-LWH), health services provide integrated Medication-Assisted and Antiretroviral Treatment Services (IMAT services). Although the IMAT comprehensive care package includes biological, social, and psychological interventions, PUD-LWH’s retention in care is suboptimal. A formative needs assessment collected qualitative information to inform adaptation for PUD-LWH of an evidence-based peer-led group psychosocial intervention that showed good clinical and psychosocial outcomes in people living with HIV.

**Methods:**

We conducted in-depth interviews with 22 PUD-LWH to understand users’ experiences with IMAT services. Healthcare providers (n=6) also participated. Thematic areas explored included perceptions of how the IMAT services process met users’ needs, experiences when attending IMAT services, including perceived benefits and challenges, and proposed strategies to overcome reported difficulties. Narrative data were analyzed using thematic analysis.

**Results:**

PUD-LWH perceived themselves as physically and mentally stable and acknowledged that IMAT had helped them re-integrate with families. Enacted stigma, out-of-pocket healthcare expenses, and limited time with healthcare providers due to an overburdened IMAT service were challenges to maintaining adherence to IMAT clinic visits and ART medication use. Participants proposed strategies to continue building awareness of IMAT services, reduce enacted stigma related to drug use, enhance client psychosocial care, facilitate the provision of skills for improving income generation for PUD-LWH, and increase the number of IMAT healthcare providers.

**Conclusions:**

This formative study reveals individual, family, and community-level facilitators and barriers to accessing and using IMAT services and IMAT services-based structural challenges. IMAT services clients by suggesting strategies to overcome structural challenges.

## Background

Illicit drug use continues to be a significant global public health problem. In 2021, an estimated one in 17 people worldwide had used a drug of abuse in the past 12 months, 23% more than a decade ago[1]. Among them, 60 million engaged in non-medical drug use, 31.5 million of whom used opiates (mainly heroin). Opioids continue to be the group of substances with the highest contribution to severe drug-related harm, including fatal overdosing[1]. Opioid use disorders (OUDs) are characterized by a problematic pattern of opioid use leading to clinically significant impairment or distress, with impairments in physical, mental, and psychosocial functioning[2]. In comparison to the general population, people with OUDs are more likely to acquire and transmit bloodborne infections like HIV and hepatitis[3]. For example, studies in Tanzania report a higher prevalence of HIV among PWUDs, ranging from 8.7% to 42%[4–7], compared to rates in the general population (4.5%)[8]. To prevent and manage HIV among PUD-LWH, the Tanzanian Ministry of Health, in collaboration with health stakeholders, implemented integrated Medication-Assisted and Antiretroviral Treatment Services (IMAT services) [9]. IMAT includes a comprehensive HIV Interventions for Key Populations package, including biological, social, and low-intensity psychological interventions[6]. Evidence shows that integrated services facilitate earlier treatment initiation, improve adherence to ART and methadone treatment, reduce stigma, and enhance client satisfaction[10–12]. IMAT also reduces costs by limiting duplication in the healthcare system and minimizing many adverse health and social outcomes related to opioid use[12]. However, retention in PUD-LWH is suboptimal [13–14], compromising clinical and psychosocial outcomes [13, 15].

The ongoing rollout of a trained peer-led psychosocial group intervention (NAMWEZA) in Tanzania’s HIV care and treatment services has positively affected retention in ART services and improved clinical and psychosocial outcomes. These include depressive symptom severity, self-efficacy, self-esteem, and perceived social support[16], as well as improvements in CD4 cell count, haemoglobin, weight, and retention in HIV care [17]. Intervened people living with HIV demonstrated confidence and abilities to utilize new skills learned, such as communicating HIV risk reduction messages to their social networks, reducing HIV-related transmission risk behaviours, and increasing their self-esteem [18]. The NAMWEZA intervention, however, was not designed for use with PUD-LWH. PUD-LWH are more likely to delay initiating ART treatment, have suboptimal service utilization, have higher rates of HIV community transmission, and have poorer HIV treatment outcomes[8, 19]. Adapting the NAMWEZA psychosocial intervention may facilitate addressing challenges to IMAT services for PUD-LWH. The formative needs assessment study aimed to collect information to inform the adaptation of the NAMWEZA peer-led psychosocial intervention for people living with HIV to improve clinical and psychosocial outcomes and retention in IMAT services. This paper describes PUD-LWHs’ experiences with receiving IMAT services.

## Methods

### Study setting

A qualitative study was conducted between March and May 2023 at the integrated medication-assisted treatment and HIV services within the Department of Psychiatry and Mental Health at Muhimbili National Hospital (MNH) in Dar es Salaam, Tanzania. IMAT services involve clinical management of HIV delivered by methadone clinic providers trained in comprehensive HIV management (HIV testing, ART initiation, managing HIV and comorbid mental and physical disorders, recording information, and a follow-up continuum)[12]. When needed, referrals to the specialized HIV care clinic are provided. The MNH IMAT clinic was selected because it serves the largest population of PUD-LWH in the Dar es Salaam region. The clinic is staffed by social workers, nurses, occupational therapists, clinical psychologists, medical officers (registrars and residents), psychiatrists, and ancillary-trained community outreach workers (COWs) who are PWUDs in recovery working as volunteers. The MNH MAT saves about 900 clients who receive daily services. About 9% (n = 81) of the current IMAT PWUDs are PUD-LWH. Given IMAT’s tertiary care location, if NAMWEZA for PUD-LWH is successfully adapted, the MNH MAT has the potential to support scale-up in the region.

**Study participants** were PUD-LWH, professional, and ancillary IMAT healthcare providers (HCPs). The former included nurses, social welfare officers, occupational therapists, clinical psychologists, and psychiatrists. Ancillary HCPs were trained community outreach workers (COWs), recovering PWUDs who volunteer at the Medication-assisted Treatment clinic, working collaboratively with IMAT professional HCPs to support service provision for PWUDs. PWUD, HCP, and COWs participants were selected purposively to ensure capturing a diversity of possible responses as follows:

1. PWUDs were selected based on sex, age (younger < 35 years; and older ≥ 35 years), HIV-positive status, adherence status to IMAT clinic visits (for both methadone and ART services as good or poor (defined as good adherence to clinic visits- either direct observes treatment (DOT) clients who attended all of last three scheduled visits and take away dose clients who attend the previous two scheduled ART drug pick-up visits; poor adherence to clinic visits - either DOT clients who missed one or more of the last three visits planned and take away dose clients who missed one or both of the last two scheduled ART drug pick-up visits)
2. HCPs’ selection criteria were cadre (nurse, social worker, occupational therapist, clinical psychologist, and psychiatrist), sex (male and female), and duration of mental health services provision experience (for six months and above).
3. Community outreach workers COWs were selected by sex (male and female).

### Data collection

We interviewed PUD-LWH (n = 22), HCPs (n = 6) trained COWs(n = 4)

The study team conducted semi-structured interviews using a locally developed interview guide based on the core components of the NAMWEZA psychosocial intervention to inform the adaptation of the NAMWEZA intervention for the PUD-LWH. The first thematic area of the interview guide explores how the IMAT services process meets the needs of PUD-LWH and experiences when attending or providing IMAT services, with probes for perceived benefits and challenges of the services. Participants were also encouraged to propose strategies to overcome reported difficulties as a step towards determining needs to address when adapting the NAMWEZA psychosocial intervention. This article focuses on the findings from PUD-LHW participants. A total of 22 interviews with PUD-LWH, lasting 40–70 minutes each, were conducted in Swahili and audio-recorded by one resident (H.M.), two registrar doctors in psychiatry (L.U. and J.K), and graduate social scientists (A.M) as data collectors. All had qualitative research training and experience in qualitative data collection. Interviewers engaged with participants for the first time during the consenting process and were trained to be respectful and non-judgmental during interviews, given the stigma usually attached to persons with substance use disorders. Furthermore, DM, PS, and MS were engaged in the field for an extended period and daily debriefed with interviewers to ensure objectivity during data collection and to support an iterative process between data collection and analysis when developing and refining code definitions and a final narrative data codebook. The interview narratives were transcribed verbatim and then translated into English. The translated transcripts were imported into NVivo-12 for coding (DM and MS) to support data analysis. The study team (identified from narrative data key themes and their subthemes to inform the thematic analysis process[20]. We followed some components of the consolidated Criteria for Reporting Qualitative Research(COREC) item checklist to enhance a systematic reporting of the qualitative methods[21]

## Results

### Characteristics of the sample population

#### Participant demographic characteristics.

Among the MAT and ART clients interviewed, 17 were male, and five were female. The median time receiving integrated M{AT and ART services was 6.5 years (IQR = 3.5–10.0 years). Under half of the IMAT clients were receiving directly observed ART treatment (9), while the remaining (N = 13) took their ART home (See Table 1).

Participants reported benefits including feeling physically and mentally healthy, enhanced psychosocial well-being and support from families and communities. Moreover, improving relationships among themselves and healthcare providers, and saving money.

*“I thank God that my health has improved since I started the Methadone and ART* (IMAT) *clinic; otherwise, I would have been dead. I was injecting* (Heroin) *many times a day; I* could not eat or take care of myself. I had wounds due to injecting drugs into *my legs…the wounds have healed, and my health is good”*

(MAT-ART Client 10, female, 40 years).

“*In comparison to the period when I was using drugs, my mental health is now stable. Before I started the care* (IMAT clinic), *my mental health was not okay…most of the day, I felt sad and was thinking of getting the drug* (heroin)”

(MAT-ART_ Client 7, male,40 years).

*“The benefit is that you regain the trust you lost before, become close to the family, and are involved again in family meetings. When they know that you have changed. If you take methadone, avoid what has been forbidden; the community does not need a torch to see the changes* (in the participants’ behaviour)*”*

(MAT-ART_COW 1, male,40 years).

*“These services (integrated MAT and ART) have benefited my mother. When I used to inject drugs, I was not at home, and I was not giving her money for food. Since I stopped using* (Heroin), *I make sure she has something to eat. I buy clothes, wear them for a few days, and give them to her to wear”*

(MAT-ART_Client 10, female,40 years).

*“Now, my mind is settled contrary to before, to the point that I care for my children. They are happy. That is a benefit of this medicine* (Methadone and ART), *and I thank God for that”*

(MAT-ART_COW2 female 36 years).

*“They* (healthcare providers) *have been telling us how they continue to be satisfied working with us as they observe us adhering to the services* (IMAT*). Moreover, they tell us that they continue to gain trust in us”*

(MAT-ART_ Client 11, male,41 years).

*“They learn (HCPs) from us. If they sit with four or five people who use drugs, they* (HCPs) *may get stories that add value or inform them on how to live with people who use drugs, as well as others with challenges in the community. They get to know our challenges, so it helps them (HCPs) in their work”*

(MAT-ART_ Client 4, male,26 years).

*IMAT services help clients save money and improve their ability to support their families*. Some clients described how their access to and use of IMAT services have helped them save their hard-earned money. For most, improved health came with the realization that they did not need to seek, purchase, and use heroin. Those with this realization were able to save money and better care for their families. The excerpts below from the narrative data are illustrative.

*“Methadone has helped me save money that I was using to buy heroin, so without a permanent source of income, I ended up doing an inhuman job to earn money and buy the drugs* (heroin) *and get out of “arosto”* (heroin withdrawal symptoms)”

(MAT-ART-client 6, male, 30 years).

“Before I joined MAT-ART services, whatever I earned, I used for drugs(heroin). Since I started the clinic, if I earn money, I can save and budget it for food and other needs without any problem”

(MAT -ART_ Client 3, male,42 years).

##### Individual-level challenges of IMAT clients:

“We (MAT and ART clients)have been told about the effects of methadone, but some of us didn’t take it seriously. They (MAT and ART clients) go out and drink alcohol; some do not use ART medication. PUD-LWH, especially those without permanent employment, face many challenges. Sometimes, it isn’t easy to get food (An implication that being able to eat and not be hungry was important when using powerful ARV drugs). Some got overdosed (Continued heroin use with methadone). So, some get treated, and others lose their lives”

(PUD-LWH is not adherent to IMAT clinic visits. Client 3 is male and 42 years old).

*“PUD-LWH have challenges* (financial challenges), *especially those who have no permanent employment. Sometimes, it isn’t easy to get food”*

(MAT-ART_ Client 4, male, 26 years).

*“Due to financial challenges, some of us (MAT and ART clients) live together and rent a house to share the rent. Some people* (MAT and ART clients) *don’t want their friends to know their HIV status. Even some who live with family don’t want family members to know their HIV status”*

(MAT-ART client 12, male, 35 years old).

##### Family and community-related challenges:

These included gaps in family and community knowledge about MAT and ART medications, lack of family support, PUD-LWH’s ability to afford out-of-pocket healthcare expenses, including daily transport to the IMAT clinic, and meeting costs for other basic needs. At an intrapersonal level, insufficient psychosocial support from the family and its surrounding community, including drug use-related attitudinal/enacted stigma, was perceived to contribute to challenges to consistent adherence to IMAT services. This may have been compounded in contexts where the family and community in which a person lives serve as their primary social support safety net.

“Some (MAT &ART clients) do not have caregivers or parents in Dar es Salaam. They come from rural areas to look for a better life and end up using heroin, so sometimes they fail to get bus fare for clinic visits, arrive late at the clinic, or don’t come at all “

(MAT-ART_ Client 14, female, 37 years old).

*Family/community enacted stigma*. In situations where family members were more easily accessible, family and community-level stigma, both of HIV status and illicit drug use, were perceived to challenge adherence to ART medication. The following excerpts from the narrative data are illustrative.

“I *took antiretroviral medications (ARV) at the clinic after the challenges I experienced at home. My stepmother stigmatized me and treated me differently. She even entered my bedroom, found my ARVs, and showed them to others. It was a challenge, so I decided to take my ART at the clinic, though it was not what I wished”*

(MAT-ART_ Client 7, male,40 years old).

“We hear people in the community talking badly about us (people who use drugs), and others (community members) will stigmatize and mock you(himself), which usually discourages me from doing so(disclosing HIV status). Therefore, I feel shy and guilty about disclosing my HIV status”

(MAT-ART_Client 12, male, 43 years old).

“We have observed the breaking up of relationships (with intimate partners) once HIV is disclosed. That’s why it is tough to disclose our HIV status. Very few can do it. We frequently experience stigma in society; people will point fingers at people living with HIV. Therefore, it is better not to say it(HIV status) at all”

(MAT-ART_ Client 14, female, 37 years old).

“Community and friends, because of poor knowledge, consider HIV scary. At the same time, some diseases are more terrifying than HIV. People stigmatize you, distance themselves, or are scared of sharing some items with the idea that you may infect them”

(MAT-ART_ Client 3, male, 42 years old).

### Structural factors

Financial constraints when accessing other health services, such as laboratory and radiological investigations (apart from services directly offered at MAT-ART that were offered free of charge), which require clients to contribute a certain amount to costs, an approach termed cost sharing, and fares for transport to the clinic were also perceived as challenges to adherence to MAT and ART services.

*“Previously, we were given medication free* at no cost to us (MAT and ART clients), *but now, due to the hospital policy, there is cost-sharing. Once you have any hospital debt, you can’t get antiretroviral medication* (ARVs). *Though ARVs are free, you must pay or reduce the debt because we use the hospital registration number whenever we come for an ARV refill, so once you have a financial obligation, you can’t access treatment.”*

(MAT-ART_ Client 14, female,37 years old).

*“Doctors are overwhelmed with other clinics for mental disorders. Some days, they come late to IMAT. Therefore, there are days that if we (clients) have many things to do to generate our income, we decide not to go* (to the IMAT clinic) *for fear that we will be delayed.”*

(MAT-ART_ Client 10, female, 40 years old).

Regarding this concern, participants emphasized the need to expand health education provided at the IMAT clinic to community storefronts working with the methadone program at Muhimbili National Hospital. For example, participants noted the importance of repeated messaging and its value for remembering things during the recovery process. The benefits of store-front-delivered health educational messages were reported to help persons remember things to avoid or adhere to. At the same time, another noted the need to extend such health education messaging to community drug addiction prevention storefronts that include issues related to living as a person with heroin addiction and HIV, including nutrition, sexual risk behaviours, HIV clinic blood tests, why adherence to ARV drugs is essential, and coping with HIV related stigma. The following excerpts are illustrative:

“Clients receiving IMAT services should be given more specific health education. It will help them(PUD-LWH) manage their health better. Ongoing health education sessions are conducted at community-based organizations collaborating with the MAT clinic. These sessions, for example, remind and help people who are receiving methadone not to mix methadone with substances like alcohol. I usually attend the community-based organizations’ health education sessions twice weekly”

(MAT-ART_COW 1, male,40 years old).

“We need to be educated more about those blood tests, and on the issue of nutrition, we should be advised on which kind of foods or fruits we should be eating to be healthy”

(MAT-ART_ Client 12, male,43 years old).

“We need to continue to be given HIV knowledge. Through that, it will help us accept our HIV status and also the knowledge about stigmatization because sometimes it happens that we stigmatize ourselves”

(MAT-ART_COW4, female,35 years old).

### Out-of-pocket IMAT expenses and possible solutions

*P*articipants proposed strategies to address the challenges they experienced with out-of-pocket healthcare expenses related to the IMAT clinic services. The excerpt below is illustrative.

“Even if they (IMAT clinic clients) have hospital debt (cost sharing for some hospital/treatment services), they should be given ARV medications and other health services. To help this, we are asking for a total exemption for all treatment-related costs, as we used to have. If this is not possible, we are asking if we have treatment debts, we would be considered to continue receiving treatment services while working to reduce the debt slowly rather than not getting treatment because ARV medications are lifesaving”

(MAT-ART_ Client 14, female, 37 years old)

Despite mentioning a need for skills training to improve income-generating activities for PWUD_LHIV, as well as interventions to address HIV and drug-related stigma as measures for improving IMAT attendance adherence for PWUD-LHIV, fewer solutions to these concerns from this sub-sample of informants were mentioned.

## Discussion

This study reports clients’ perceived benefits and challenges of integrated MAT and HIV (IMAT)services and strategies to overcome these challenges. It is part of a formative study to inform the adaptation of a peer-led psychosocial intervention for the PUD-LWH. The adapted psychosocial intervention aims to improve clinical and psychosocial outcomes and retention in care for the PUD-LWH receiving integrated MAT and HIV services.

The participants reported feeling physically and mentally stable as they received integrated treatment for substance use disorders and HIV infection. The perceived health benefits mirror the broad evidence on outcomes of a comprehensive management approach for HIV prevention and treatment for people who use drugs [8]. The management approach emphasizes the need for an integrated, multidisciplinary intervention approach to improve the well-being of PUD-LWH. Moreover, the perceived well-being of PWUDs receiving the integrated MAT and HIV care may reflect the possible outcomes of methadone maintenance treatment among PUD-LWH[22–23].

Psychosocial interventions as part of the comprehensive packages of care of integrated MAT ART services were acknowledged to enhance participants’ essential life skills, reflecting previous findings in Tanzania conducted among the general population living with HIV receiving HIV care and treatment in a study region[16–18,24]. Such skills helped PWUD-LHIV improve their intrapersonal skills and relationships with family, community members, and HCPs. Moreover, they facilitate expected and meaningful community engagement. This is in keeping with the recommended standard of care intervention packages for people who use drugs [1], which emphasizes the need to improve the psychosocial well-being of PWUDs to facilitate their recovery.

As in other studies among PWUDs (25–27), this study also reports that participants experienced perceived individual, family, and structural challenges. These challenges interfere with their ability to maintain adherence to IMAT and ART medication use, perhaps mediated through enacted stigma following disclosure of HIV status[27]. Therefore, they perceived that they risked experiencing a double stigma due to their drug use behaviour and being a person living with HIV infection[27]. Moreover, similar to findings from a Tanzanian sample of PUD, participants reported challenges due to financial constraints, including transport costs to attend IMAT clinics and the cost of essential needs such as food[28]. Structural challenges included participants’ observations of overburdened IMAT healthcare providers and the need to pay out of pocket for their healthcare services.

Nevertheless, participants proposed strategies for addressing some of their experiences and reported challenges as integrated MAT and ART service users. This includes continuing to build awareness of IMAT in communities and developing strategies for enhance psychosocial care for IMAT clients. Moreover, they propose a need for IMAT services-based interventions to address HIV and drug-related stigma and increase the number of healthcare providers in IMAT services. While the need to facilitate the provision of skills for improved income generation in PUD-LWH was an important challenge, less contributions to strategies were made. Most of these proposed interventions reflect the UNAIDS-recommended strategies aimed to facilitate and improve the clinical and psychosocial outcomes of PUD-LHIV [8].

### Implications

Engaging people with lived experience with illicit drug use and HIV in the design, development, planning, and implementation of targeted interventions could facilitate buy-in and meaningful engagement, thereby enhancing the interventions’ feasibility[29]. Our study explores the experiences of integrated MAT and ART IMAT service clients to inform adaptation of an evidence-based peer-led psychosocial intervention. The engagement of primary stakeholders during adaptation may facilitate feasibility, piloting, and implementation and improve the potential for optimal clinical and psychosocial outcomes of an improved IMAT clinic intervention.

### Limitations

The generalizability of the study may be limited to lower-level healthcare facilities such as dispensaries, health centers, and district hospitals, as this study was conducted at a national tertiary hospital, where healthcare services are provided with a specialized and multidisciplinary approach. We did not interview the family/relatives of the MAT and ART clients to explore their experiences with the integrated MAT and ART services. We acknowledge that they could have views regarding IMAT services offered to their relatives. Lastly, the study used a qualitative method that limited generalizability. However, our findings have merit as a first stage towards identifying, from clients’ perspectives, the challenges and potential strategies for improvements in IMAT service outcomes.

## Conclusion

This formative study reveals the benefits of individual and family support for adherence to IMAT services while uncovering individual, family, and structural challenges from users’ perspectives. These findings are valuable for informing the adaptation of peer-led psychosocial interventions for PUD-LWH and for developing and piloting an improved comprehensive care package for IMAT.

## Figures and Tables

**Table 1:** Individual interview participants and their socio-demographic characteristics in Dar es Salaam, 2023.

Characteristics	Male n (%)	Female n (%)	Total N
Clients receiving integrated MAT and ART	17 (77)	5 (23)	22
Education attained			
None formal	3 (100)	0	3
Basic Primary Level (Total Seven years)	10 (83)	2 (17)	12
Ordinary Secondary (Total 11 years)	4 (57)	3 (43)	7
Advanced Secondary Level (Total 13 years)			
College/University Education			
Marital Status			
Living with a partner (formal or informal)	8 (83)	3 (27)	11
Widow/widower			
Divorced/separated	2 (50)	2 (50)	4
Single and never lived with a partner	7 (100)	0	7
Living circumstances			
Lives alone in rented accommodation	1(100)		1
Lives with friends in rented accommodation	-	-	
Lives with spouse and child/children in rented accommodation	3 (50)	3 (50)	6
Lives with family of origin in rented accommodation	-	-	
Lives with family of origin in a family-owned home	9 (90)	1 (10)	10
Lives with spouse and child/children in an owned home	1 (100)	-	1
Lives alone in Asylums/no known home	3 (75)	1	4

## Data Availability

The article summarizes all the data used to write this paper. If the study narrative data is needed, please contact the corresponding author.
